# A qualitative process evaluation of training for non-physician clinicians/associate clinicians (NPCs/ACs) in emergency maternal, neonatal care and clinical leadership, impact on clinical services improvements in rural Tanzania: the ETATMBA project

**DOI:** 10.1136/bmjopen-2015-009000

**Published:** 2016-02-12

**Authors:** David R Ellard, Aloisia Shemdoe, Festo Mazuguni, Godfrey Mbaruku, David Davies, Paul Kihaile, Senga Pemba, Staffan Bergström, Angelo Nyamtema, Hamed-Mahfoudh Mohamed, Joseph Paul O'Hare

**Affiliations:** 1 Warwick Clinical Trials Unit, Division of Health Sciences, Warwick Medical School, The University of Warwick, Coventry, UK; 2 Ifakara Health Institute, Dar es Salaam, Tanzania; 3 Educational Development & Research Team, Warwick Medical School, The University of Warwick, Coventry, UK; 4 Tanzanian Training Centre for International Health, Ifakara, Tanzania; 5 Department of Public Health Sciences, Karolinska Institutet, Stockholm, Sweden; 6 Division of Metabolic & Vascular Health, Warwick Medical School, The University of Warwick, Coventry, UK

## Abstract

**Objectives:**

The Enhancing Human Resources and Use of Appropriate Training for Maternal and Perinatal Survival in sub-Saharan Africa (ETATMBA) project is training non-physician clinicians as advanced clinical leaders in emergency maternal and newborn care in Tanzania and Malawi. The main aims of this process evaluation were to explore the implementation of the programme of training in Tanzania, how it was received, how or if the training has been implemented into practice and the challenges faced along the way.

**Design:**

Qualitative interviews with trainees, trainers, district officers and others exploring the application of the training into practice.

**Participants:**

During late 2010 and 2011, 36 trainees including 19 assistant medical officers one senior clinical officer and 16 nurse midwives/nurses (anaesthesia) were recruited from districts across rural Tanzania and invited to join the ETATMBA training programme.

**Results:**

Trainees (n=36) completed the training returning to 17 facilities, two left and one died shortly after training. Of the remaining trainees, 27 were interviewed at their health facility. Training was well received and knowledge and skills were increased. There were a number of challenges faced by trainees, not least that their new skills could not be practised because the facilities they returned to were not upgraded. Nonetheless, there is evidence that the training is having an effect locally on health outcomes, like maternal and neonatal mortality, and the trainees are sharing their new knowledge and skills with others.

**Conclusions:**

The outcome of this evaluation is encouraging but highlights that there are many ongoing challenges relating to infrastructure (including appropriate facilities, electricity and water) and the availability of basic supplies and drugs. This cadre of workers is a dedicated and valuable resource that can make a difference, which with better support could make a greater contribution to healthcare in the country.

**Strengths and limitations of this study**

- The study provides an insight into the challenges faced by the cadre of workers who work in rural Tanzania.
- Upskilling this cadre of health workers could have a positive impact on key health outcomes.
- It is our belief that as the trainees share their new skills and knowledge, the impact will grow.
- A limitation is that these were one-off interviews.

## Background

The Enhancing Human Resources and Use of Appropriate Training for Maternal and Perinatal Survival in sub-Saharan Africa (ETATMBA) was a European commission (FP7) funded project.^1^ In rural Tanzania, it provided advanced clinical and leadership training (between November 2011 and June 2014) to non-physician clinicians (NPCs) or nowadays associate clinicians (ACs). The concept ‘non-physician clinician’ is already obsolete and actively abandoned by this cadre in Africa. There is now an active professional network called ANAC (African Network for Associate Clinicians) representing the interests and needs of assistant medical officers (AMOs)/COs across several sub-Saharan countries.^2^ In Tanzania, they are often referred to as clinical officers (CO) or AMOs (more experienced COs who have received some additional training), and they provide emergency obstetric care (EmOC).

There is inequity in the range of health worker skills with many low-income countries, particularly in Sub-Saharan Africa, having too few specialist doctors such as surgeons, obstetricians and anaesthetists, relative to the health needs of their populations.^3–5^ This widespread crisis in the health workforce is affecting the realisation of the health-related millennium development goals (MDGs), particularly, MDG 4, reducing child mortality, and MDG 5, improving maternal health.^6^ ^7^ About 800 women die from preventable causes of pregnancy or childbirth-related complications around the world every day. In 2013, 289 000 women died during and following pregnancy and childbirth. Almost all of these deaths occur (99%) in low-resource settings; more than half of these deaths occur in sub-Saharan Africa and almost one-third occur in South Asia. The sub-Saharan Africa region alone accounted for 62% (179 000) of global deaths followed by Southern Asia at 24% (69 000).^8^ In Tanzania, assessments of maternal and newborn child health show that approximately 7900 women die each year from complications of pregnancy and childbirth.^9^ Contributing factors include: limited access to health services, especially emergency maternal and newborn care, lack of appropriate referral systems, shortage of skilled healthcare workers and lack of infrastructure including essential equipment and drugs.^10^ Indeed, even if services are available, they can prove to be of poor quality.^11^ Addressing the worldwide skilled healthcare workforce crisis is an ongoing problem. Countries like Tanzania as far back as the 1960s adopted the NPC/AC task shifting/sharing model and it is this cadre of health workers who are in the frontline of innovative healthcare, in particular CEmOC, very often in rural and remote areas.^12^

The ETATMBA project was a 3-year programme of training for NPCs/ACs focusing on upgrading the knowledge, skills and clinical leadership related to CEmOC in Tanzania and Malawi. The project's goal was to determine whether upskilling NPCs/ACs in maternal and neonatal care and clinical leadership can impact on clinical services improvements in rural Tanzania. More details about the training can be found on the project website (see web appendix for more information).^1^ The evaluation of the ETATMBA programme in Tanzania involved a before and after study, a survey of health facilities and a qualitative process evaluation. The following sections describe the methodology and results of the qualitative process evaluation.

## Methods

### Design

A qualitative process evaluation (interviews) exploring the implementation and acceptability of the ETATMBA training programme from the perspective of a number of stakeholders including the trainees, the trainees’ district medical officers (DMOs), colleagues (whom they have cascaded ETATMBA skills to) and their trainers. Evidence of changing clinical practice was also explored.

### Research team

The research team was mainly composed of Research Scientist from the Ifakara Health Institute (IHI) Dar es Salaam, Tanzania. The primary data collection team consisted of two local research assistants (AS and FM) based at IHI. Both of the research assistants had great experience in qualitative research. The principal investigator at the IHI (GM) gave support to the local team while management/oversight was provided by DRE from Warwick, UK.

### Participants

During late 2010, early 2011, 36 trainees (AMOs and nurse midwives/nurses (anaesthesia)) were recruited from districts across Tanzania and invited to undertake the ETATMBA training programme (see web appendix for more information).^1^ While there was some attrition (eg, withdrawal from the training), the remaining trainees represent the sample from which we invited all to participate in evaluation interviews. In addition, we identified a number of DMOs and cascadees to be involved in interviews from facilities where trainees had been working. A cascadee was a nurse, midwife, AMO or CO with whom ETATMBA trainees shared their ETATMBA skills and knowledge. We also purposively invited a number of the local training facilitators to be interviewed.

### Procedure

#### Interviews

As a first step, the researchers identified the facilities where trainees were based. A letter of invitation including an information sheet and a copy of a consent form was then sent via email to all trainees from the IHI. Second, letters and information to the DMOs were similarly emailed. The letter had two purposes: first to inform them about our research in general, and second to invite them to participate. A copy of the consent form was included.

In recognition that Tanzania is a very large country and road access is at times problematic, the research team arranged a ‘grand tour’ of all of the included districts and health facilities. This was undertaken in January/February 2014. This limited the opportunities to carry out interviews with everyone. In all districts the researchers invited all of the available trainees, cascadees and DMOs for interview.

The research team developed an interview guide prior to the ‘grand tour’ that was used in all interviews. It was designed to cover the whole experience surrounding the training and specifically pressed for actual examples as evidence of changing practice. It was not enough for the trainee in the interview to just to say ‘yes’ or ‘no’ when questioned about the training; we encouraged them to provide specific examples. The semistructured Interviews were carried out at or near the health facilities at mutually agreeable times and held in a quiet private room during the researcher's visit; confidentiality was assured.

The IHI researchers conducted most of the interviews in Kiswahili to ensure no loss of meaning in expressions. English is officially the second language in Tanzania but it is commonly spoken and all of the trainees have good levels of English; however, it was found that they were more comfortable using Kiswahili. There were no formal inclusion exclusion criteria for this evaluation as we were targeting specific populations. Those outside these groups were not invited.

### Data analysis

Interviews were digitally recorded, subject to the permission of each participant and, where transcribed, verbatim. Recordings were stored in a secure digital environment accessible only to members of the research team. Participants were not identified by name; instead, a participant code number was used to identify transcripts. Data were analysed using the Framework method described by Ritchie and Spencer^13^ and Pope and Mays^14^ (see box 1).

Box 1 Overview of the framework method of qualitative analysis*

- Data familiarisation: reading of complete interview transcripts, listening to original audio-recordings and use of field notes;
- Identifying a thematic framework: key issues, concepts and themes are identified and an index of codes developed;
- Indexing: whereby the index generated through identification of the thematic framework is applied to all data;
- Charting: a summary of each passage of the text is transferred to a chart to allow more overall and abstract consideration of index codes across the data set and by each individual;
- Mapping and interpretation: understanding the meaning of key themes, dimensions and broad overall picture of the data and identifying and understanding the typical associations between themes and dimensions;
- The charting process provides an opportunity to code data from numerous vantage points, by demographic factors, such as gender or age, by personality characteristics, such as looking specifically at people who are highly anxious compared to those who are not, or by medical aspects, such as those with diabetes compared to those without.
- The charting process provides an opportunity to code data from numerous vantage points, by demographic factors, such as gender or age, by personality characteristics, such as looking specifically at people who are highly anxious compared to those who are not, or by medical aspects, such as those with a particular condition compared to those without.

* Adapted from Ritchie and Spencer.^13^

The computer package NVivo V.10 was used to facilitate this process. The data were coded by the local researchers (AS and FM). Researcher bias was minimised through regular cross-checking of data and findings by the members of the research team DRE in the UK provided validation of themes. We note here that analysis of the process evaluation data (the interviews) was carried out before and without the knowledge of results from the quantitative studies (which will be reported elsewhere).

Quotations are used as exemplars of themes. Each quotation has an identifier. The ‘ETATMBA trainer’ is identified thus, as are the three Obstetricians. Trainees are identified as T, followed by their profession, for example, NPC/AC, NA (anaesthetic nurse), NMW (nurse midwife) and finally a number (1–27). Cascadees (those who have received training from our trainees) are identified by CA and a number (1–12). DMOs and doctors in charge are identified as managers (MA) and a number (1–5).

### Ethical approval

The study was reviewed and approved by the Biomedical Research Ethics Committee (BREC) at the University of Warwick, UK (REGO-2013-572) and The National Institute for Medical Research, Institutional review board, Dar es Salaam, Tanzania (no.35, dated 9 March 2012).

## Results

Thirty-six received the ETATMBA training including 19 AMOs, one CO, and 14 nurse midwives (NMW) and two nurses (anaesthesia). During the project period, one AMO and one NMW left the programme to pursue other interests and one NMW died. Thus, attrition at the end of the programme was around 8%.

Trainees were based in health centres and district hospitals across Tanzania in rural or very remote areas. Trainees were recruited from health facilities where an ‘upgrading’ was agreed on. The upgrading, while not part of the ETATMBA project, was an ongoing piece of work provided by the government and other agencies. The ‘upgrading’ included the provision of infrastructure like operating theatres and as such would allow the ETATMBA trainees to put into practice their new skills. However, the reality was that of the 33 trainees who completed the programme, only 19 returned to the place from where they were selected and seven of these returned to facilities that had not been upgraded or where upgrading was still in process. Fourteen trainees did not return to the facility where they were recruited as the facilities had not been upgraded. Most of these (10/14) were returned to district hospitals in the area they came from. Table 1 below gives an overview of where the trainees were based and the type of facility they worked in. This also notes the availability of an operating theatre in the facility as this is one of the key things that was to be upgraded (note: upgrading of facilities was not part of the ETATMBA project but was ongoing work with the Government and other funding agencies).

**Table 1:** Health facilities where the Tanzanian ETATMBA trainees were based in 2013

	District	Name of facility	Operating theatre	CEmOC or BEmOC	Trainees, (n)
1	Bukombe	Bukombe District Hospital	Yes	CEmOC	1 AMO
2	Bukombe	Uyovu Health Centre	No	BEmOC	1 AMO, 1CO
3	Geita	Nzela Health Centre	Yes	CEmOC	1 NMW, 1 Nurse
4	Geita	Katoro Health Centre	No	BEmOC	1 AMO, 1 NMW
5	Inyonga	Mamba Health Centre	Yes	CEmOC	1 NMW
6	Karambo	Matai Health Centre	No	BEmOC	1 AMO, 1NMW
7	Liwale	Liwale District Hospital	No	CEmOC	2 AMOs
8	Meatu	Mwandoya Health Centre	No	BEmOC	1 AMO, 1 NMW
9	Mpanda	Mpanda District Hospital	Yes	BEmOC	1 AMO, 1 Nurse
10	Nachingwea	Nachingwea District Hospital	Yes	CEmOC	2 AMOs
11	Nkasi	Kirando Health Centre	Yes	CEmOC	2 AMOs
12	Nyanghwale	Nyanghwale Health Centre	No	BEmOC	1 AMO, 1 NMW
13	Nyanghwale	Kharumwa District Hospital*	Yes	CEmOC	1 AMO, 1 NMW
14	Ruangwa	Ruangwa District Hospital	Yes	CEmOC	1 AMO, 1 NMW
15	Sumbawanga	Laela Health Centre	No	BEmOC	1 AMO, 1 NMW
16	Chato	Chato District Hospital	Yes	CEmOC	1 AMO, 1 NMW
17	Lindi	Nyangao Mission Hospital†	unknown	CEmOC	2 NMWs

*Upgraded to a district hospital between 2011 and2013.

†This hospital was not visited, so it is not included in the analysis.

AMO, assistant medical officer; BEmOC, basic emergency obstetric care; CEmOC, comprehensive emergency obstetric care; ETATMBA, The Enhancing Human Resources and Use of Appropriate Training for Maternal and Perinatal Survival in sub-Saharan Africa; NMW, nurse midwife; nurse, nurse/anaesthetics.

In total, 27/36 trainees, 12 cascadees, 5 managers and 3 ETATMBA obstetricians were interviewed. The qualitative interviews explored the following themes around the ETATMBA training, including: the selection of trainees, delivery of the training, relationships between NPCs/ACs and medical doctors. Implementation of training into practice, support for implementation, challenges, impact of training, sustainability and recommendations. Quotations are provided in Panels.

### 

#### Selection of trainees

The ETATMBA trainees were invited from diverse locations across Tanzania to attend the training with a plan to recruit a pair made up of a senior (AMO) and a junior NMW (anaesthesia) from a health facility. Selection was carried out by the ETATMBA project obstetricians in Tanzania in collaboration with the Ministry of Health and local District Medical Officers (DMOs) (box 2). Selection of facilities was a pragmatic one but requiring the availability of health workers with the required experience and willingness to participate, together with the agreement with the DMO and either that a facility was or had been upgraded (see online supplementary appendix).

Box 2 **Selection of trainees and delivery of the training**

-
  …ETATMBA objectives are AMOs and NMWs from health centres that have been doing surgery or they were planning to build theatres, these were the criterions. And if there were none in the district let us say Lindi or we plan to build one. So we decided to take from district hospitals because actually in district hospitals even the regions AMOs do run maternity wards. (Obstetrician 3)
-
  This was a modular training so we called participants from various selected districts; the training was convened in one training unit centre. These participants were mainly AMOs and NMWs, AMOs were clinical people and NMWs were called in to be trained as nurse anaesthetists. (Obstetrician 1)
-
  Some of these guys have been trained 20 years ago they do not know new approaches of treatment so if they are taught that this is how such kind of problem can be managed they will be more interested because for them this is new knowledge, they were more attentive, they took some notes, they asked questions. For us this was very positive. (Obstetrician 1)
-
  …we were well received and our teachers cherished and loved us. (T.NA 4)
-
  …we had good accommodation, we had necessary learning materials, we could easily access internet freely and we could get reference books. (T.NPC 18)
-
  …we were promised that we shall be provided with laptops but that never happened. Only trainers were using laptops. (T.NPC 5)

#### Delivery of the training

Training was delivered using a competence-based education curriculum where the emphasis was on ‘hands on’ training. The training period lasted for 3 months. Trainers reported a very positive attitude towards their trainees. They stated that most of the trainees were health workers who had many years’ experience, yet the training was still important as they were updated with new knowledge and skills which added value to what they knew prior to the training.

Trainee's perceptions of the training were generally very good with a majority saying that they liked the training.

Most of the trainees reported that the accommodation and learning environment was very conducive to learning. Some did, however, have issues with accommodation, food and allowances. One interviewee noted that they were promised laptops and these were not provided (box 2).

#### Interaction between trainees and MDs

Interestingly, there were varied perspectives on the relationship between trainees and medical doctors. The majority of trainees, cascadees and managers (in both the district hospitals and health centres we visited) reported that the relationship between trainees and MDs has been positive both before and after the training.

The trainers had different responses on this; they revealed that tension among the two groups is historical and it has existed for a long time.

However, trainers pointed out that ETATMBA training had dissolved the tension and brought cohesion across the two groups; medical doctors did feel that the AMOs performed better after having been updated with new skills and knowledge, suggesting that task shifting in the area of CEmOC is a feasible and acceptable approach (box 3).

Box 3 **Interactions between trainees and MDs and expectations post training**

-
  …now they are working together collaboration has been intensified. (CA 10)
-
  ….relationship between them is a little bit complex because you see the AMOs, most of them have worked for a long time and they have a vast experience especially in surgery whereas the Medical Doctors are fresh from universities actually their experience is very different so, they feel very embarrassed when they are with AMOs… they are called medical doctors but they can't do a lot of things that an AMO can do. (Obstetrician 3)
-
  Instead of bringing competition it brought more cohesion in the sense that the doctors are more confident that the AMOs are doing operations properly. (Obstetrician 1)

#### Expectations

The majority of trainees reported that they were disappointed because their expectations had not been met. They were informed that after the training they would be assigned to work in upgraded health centres where they would be able to implement knowledge and skills they gained at the training (box 3).

#### Implementation of training into practice (cascading, support received and challenges)

In regard to implementation of training into practice or what is commonly referred to as learning transfer, most of the trainees reported that they were now managing various cases on their own, only calling for support or help if unforeseen problems were encountered. They noted that they were now more confident in performing certain procedures including performing caesarean sections, management of PPH, (pre-)eclampsia, how to position and resuscitate babies competently. They noted that the training had updated their knowledge and skills.

The interviews revealed that cascading of the training was taking place and was changing practice at their place of work, indeed even suggesting that they were supporting junior doctors who lacked experience (box 4). Also, other team members were making better use of tools like the partograph which the trainees had taught them to use more effectively.

Box 4 **Implementation of training into practice (including support and challenges) and Application of clinical leadership skills**

**Implementation of training into practice**

-
  Training has been of a great help to me, I now work in theatre with more confidence than previously. I now understand how to differentiate the anaesthesia medicines and recognising a patient that should or should not be provided with such medications. (T. NMW 13)
-
  …in neonatal resuscitation we were taught differently from what we knew. We used to resuscitate a baby with breathing problem using adrenaline medicine. In school we were not taught to use any medicine for resuscitation, it was if you see that a baby can't breathe properly to put him/her on oxygen machine. (T.NA 27)
-
  They taught us how to record partograph to understand that now this woman goes to an action line and be able to take action earlier before she gets other complications. It enables us to be keen in filling of partographs. (CA 1)
-
  ..although there are still some few challenges in filling of partographs especially among the medical attendants, these are people who stay in labour room they conduct a lot of deliveries, some of them are filling well, some are not but we keep on instructing them slowly. (T.NPC 17)
-
  …they cascaded the knowledge to most health workers in maternity ward and they have really assisted junior doctors who are fresh from school but they are not experienced. (MA 1)
-
  …it has assisted me to be innovative for example we have few operating gowns at the hospital. One day I had to use drapers (curtains) as a surgery gown. There was a woman who was delayed to come to the hospital for delivery when they rushed her here she was profusely bleeding. When she arrived here we found there were not any operating gowns. I was with my colleague we went together for ETATMBA training as an anaesthetist we had to put on drapers and performed surgery and both a mother and a baby survived. (T.NPC 15)

**Support (received)**

-
  ‘After training my health facility in-charge was coming to see how I perform but my DMO never visited to see how I work.’ (T.NPC 2)
-
  ..he calls me (obstetrician/trainer) and asks me if whether I came across any emergency cases; if I have one I tell him. I can call him even at midnight and he is always happy to assists (if I have a problem). (T.NA 4)
-
  …they assisted us getting solar power here it is the MP X, she went to the Ministry of Energy and Minerals and ensure that we get solar power although we can't use for generator at night. (T.NPC 8)

**Challenges**

-
  ‘…for example you conduct C-section you should ensure the availability of blood for transfusion, you go to a laboratory there is no blood bags, you ask yourself, what do I do? ‘(T.NPC 21)
-
  …there are 5 old houses here; they are very old with various insects including bees and bats. Health workers are not ready to come and work here, (T.NPC 10)

**Application of clinical leadership skills**

-
  ‘I have a good relationship with my DMO. When I came here I found only 1 CO after seeing increase of patients who comes for services I asked the DMO to bring another CO, he promptly responded now the workload is a bit reduced because I have adequate staff. I also get sufficient support from community leaders… (T.NPC 16)

Although there were no formal supportive supervision arrangements made after the training, the majority of trainees said that they had been frequently receiving support from one of the ETATMBA trainers whom they identified as their mentor. All of them reported that at one point of time the trainer had been calling to ask them whether they needed any kind of clinical advice, which he was always ready to support. A few trainees were physically visited to encourage them to use the new skills gained.

The majority of trainees in districts noted that they had received adequate support from the district level, particularly DMOs, except in one district where trainees were disappointed with a DMO who was not supporting implementation of the training. The DMO was a new appointee at the facility and the trainees felt that he was against surgery conducted by AMOs in health centres.

At a health facility level, the majority of trainees said that they had been receiving support from upper to lower levels, that is, health facility managers (health facility in charge, colleagues at their levels and junior staff). At the community level, the majority of trainees said that their community leaders had been very supportive (box 4).

Most of the challenges that were mentioned by trainees were clinical challenges, particularly system constraints including lack of medical supplies and equipment (eg, vacuum equipment, blood bags). The Medical Store Department (MSD), a Ministry of Health and Social Welfare (MOHSW) department with responsibility for maintaining supplies, is often blamed for failure to bring medicine to health facilities in a timely manner. In addition, lack of transport facilities (ambulances for referrals), lack of infrastructure (theatre room, room for neonate resuscitation), poor electricity and water supplies are all noted as challenges. Electricity supplies are reported as sporadic and in some centres where there are generators it is noted that these are poorly maintained. There were also reports that generators were damaged during fires and also lack of fuel to run them if they were operational.

Trainees also talk about the provision of housing for them near to the health facility. Houses were often of very poor quality or indeed no provision was made.

#### Application of clinical leadership skills

During the training, trainees were exposed to key leadership issues so as to enable them to overcome some of the challenges related to their new roles. One trainee felt that the training had given him confidence to raise issues with senior staff and others and work towards finding solutions to challenges (box 4).

#### Impact of training

A large majority of trainees, cascadees and managers said that one of the most notable impacts of the training was the much improved quality of EmOC. Also, neonatal resuscitation procedures and skills and knowledge of how to deal with obstetric emergencies have had an impact.

A number of the trainees describe how they believe that women are now attending their facility because of their enhanced training with one even suggesting that they come to his health centre when the district hospital is nearer. One also feels that home deliveries have reduced in the area (box 5).

Box 5 **Impact of training**

-
  …maternal deaths have been reduced, I remember when we came back there were 29 deaths, this year we had only 12 deaths. (T.NPC 15)
-
  …previously when you looked at new-borns deaths, most of them were dying soon after birth. Maternal deaths also were rampant in previous years. (T.NPC 21)
-
  …if you go through our books maternal mortality rate has been reduced. We once had 101 maternal deaths it was then reduced to 4 deaths, last year we had only 4 deaths (if I'm not mistaken) maternal mortality so, I do appreciate the training (MA 1)
-
  The training has brought a big impact. We can now assist new born with difficult to breath, Now we can assist such children as we integrate with Help Baby to Breath programme (HBB) we offer the best service for new-borns. (CA 10)
-
  …for example for obstetric emergencies like eclampsia previously we were using ketamine which is very risky to patients but now we use spinal anaesthesia and it has significantly reduced maternal and child mortality. (MA 5)
-
  …number of mothers who come to the hospital for deliveries have increased, previously we had 40 deliveries now we have almost 100 deliveries. (T.NPC 21)
-
  Yes, most mothers now do come here for delivery. Home deliveries have also decreased, after starting theatre procedures including C-sections, mothers have stopped going to the TBAs, and they now come here for deliveries. (CA 5)
-
  …some mothers and babies were dying during long journeys to hospitals from remote health centres like ours. After the training we have been able to assist mothers (with appropriate drugs and treatment)who had to travel these long distances (e.g.1130 kms) and we are no longer experiencing these deaths; I have managed several of these cases. (T.NPC 21)

#### Recommendations and sustainability

In the later parts of the interviews, we explored the recommendations the trainees and trainers may have about the ETATMBA programme. In clinical training, it was suggested that more time be given to some training topics including the management of obstetric and birth complications, surgery, anaesthesia, completion of partograms and leadership.

During the interviews, we elicited trainers and managers’ perspectives about the sustainability of the training. Most seemed optimistic that the training can be sustained but certain strategies need to be set. One manager stated that there is a need to integrate the training in their ‘local’ planning (box 6).

Box 6 **Recommendations and sustainability**

**Recommendations**

-
  ‘…more emphasis to be allocated in training of clinical skills including performing of C-sections. Trainers should not assume that all trainees can perform at the same level. (T.NPC 1)
-
  …more time to be allocated obstetric complications topic including PPH, eclampsia and antepartum haemorrhages severe anaemia in pregnancy. (T.NPC 2)
-
  …more time to be allocated for topic on leadership because we came here without any knowledge on leadership matters. (T.NA 9)

**Sustainability**

-
  …we need to put this in our Council Comprehensive Health Plan (CCHP) because anything that is donor oriented can't be sustained, so when a donor leaves everything ends there. So we shall include this training in our CCHP. (MA 2)

## Discussion

The attainment of the objectives of this process evaluation, exploring the implementation of the ETATMBA training in Tanzania, has been very enlightening and successful. A high proportion of the trainees were interviewed and therefore contributed to this evaluation. In addition, we were able to interview a representative sample of managers and cascadees.

It is clear that the initial plan to return trainees to new and upgraded facilities post-training was not carried out. However, this was outside the control of the ETATMBA project. Some trainees returned to health facilities where their new-found skills could not be used effectively. Other trainees were sent to district hospitals.

The training was generally well received by the trainees, although it became apparent throughout the interviews that while the questions were about their experiences as a result of ETATMBA, the answers reflected historical feelings about issues that were beyond the scope of ETATMBA such as poor housing provision, motivation/retention, lack of infrastructure and inadequate supplies including drugs. In a recent study conducted in rural health facilities in the country, two dimensions of health workers’ environment, namely infrastructure and supportive interpersonal work environment, were found to a large extent to explain much of the variation in satisfaction among rural health workers.^15^

The teaching resources provided were generally seen to be adequate but it was noted that laptops were not provided, and this restricted their potential access to electronic resources. While it was never planned within ETATMBA to provide laptops, a rumour did start among the trainees and when none appeared, there was some discontent early on. Similarly, there were comments about food and allowances. These were all just minor, indeed normal, niggles that occur within programmes like this. The trainees were paid allowances at a rate similar to that paid for other formal training.

Unlike other CEmOC training programmes, this particular training had a component of clinical leadership which aimed at building trainees’ capacity to mobilise and align resources in their workplaces using their own initiative. In the Malawi arm of the ETATMBA project, the trainees reported that the leadership training was a new experience they valued highly, and indeed there was considerable evidence that it helped them in their role.^16^ Here, in Tanzania, leadership training was also a new experience, but there was only limited evidence of its use. The working relationship between trainees and the medical doctors seems to have improved. Indeed, we found evidence that junior doctors are now drawing on the expertise of some of the trainees.

There is also evidence that the training has been implemented into practice and that the planned cascading of skills and knowledge to colleagues has taken place, which is very encouraging. The sharing of knowledge and skills was well received and appears to have brought about more team working. The example from one of the medical attendants about the correct use of partographs suggests a better working relationship, within teams, in the facilities. Support for the training at the facility level was generally good, but a small number of trainees did meet some resistance to them doing surgery. There are still some that view this cadre as not being skilled enough to carry out the procedures and practice in the ways they do. Supervision, provided from a distance, was seen as good with the trainees feeling they could, if needed, talk to a trainer. However, most would have liked some more visits from the team. Practically, this proved difficult with a limited budget, distance and remoteness being the main barriers.

Most of the trainees provided good evidence that the training was having an effect in their facility, thus giving us a picture that suggests that the ETATMBA project may have had a positive impact on patients’ lives.

Recommendations suggested by trainees reflect just small changes to the current training curriculum programme but also reflect the challenges faced on a day-to-day basis by this cadre. The impact of the training can only be sustained if the infrastructure (eg, facilities, electricity and running water), supplies and drugs are made available. There was also considerable disappointment when trainees found that their facilities had not been upgraded as planned, or indeed facilities were upgraded but either not working or of poor standard precluding them from being used as proposed. Indeed, the motivation for any health worker to work in remote rural areas without proper provision of housing, infrastructure and support is an ongoing problem, one which needs to be addressed.^7^ ^17^

This study has a number of limitations, not least that interviews were carried out only once with each participant. In a similar study in Malawi, we carried out interviews on a number of occasions to get a greater understanding of the process.^16^ Our interviews required the trainees to reflect on the whole process. However, we do have a large volume of data from the trainees and other stakeholders. Also, owing to time constraints, we did not include users of the services (community). There is also the possibility of confounding factors. There are many health-related initiatives being delivered, often by NGOs, across countries like Tanzania; indeed, an interviewee in this evaluation mentions the ‘helping baby's breath’ initiative. Facility deliveries are not always associated with improved maternal health outcomes, and a recent review found higher mortality for women delivering in health facilities in Sub-Saharan Africa.^18^

Our findings support those from an earlier study that suggests that the training has had an effect on maternal mortality in the facilities.^19^ We also now know that in one district in Tanzania our trainees have had the confidence, post training, to talk to the local government and the MOHSW and have been instrumental in encouraging them to upgrade more health facilities to provide CEmOC.^20^ Evidence from our quantitative study support our positive findings here.^21^ In many respects, our findings here match those of the ETATMBA trainees in Malawi. In both cases, the trainees report challenges relating to resources, but there is also some evidence of a positive impact of the training on health outcomes.^16^ However, similar to the ETATMBA results in Malawi the full effect of the training may take a year or so to be realised as the skills and knowledge are cascaded. Indeed, limitations to staff performance in this context are not new as has been found in similar studies in the country.^22^ This cadre of health workers, provided with high quality training like ETATMBA, can make a difference to maternal and neonatal health, and in this aim they have considerable support.^5^ ^23–25^

In conclusion, the ETATMBA training programme was successfully implemented in Tanzania. Any lasting impact of the upskilling of this cadre may be dependent on some greater recognition of their value. Given the proper recognition and the tools to do the job, this hardworking and dedicated cadre of health workers will benefit the health and welfare of citizens in Africa.
